# Little effect of seasonal constraints on population genetic structure in eusocial paper wasps

**DOI:** 10.1002/ece3.366

**Published:** 2012-09-14

**Authors:** Thibault Lengronne, Ellouise Leadbeater, Solenn Patalano, Stephanie Dreier, Jeremy Field, Seirian Sumner, Laurent Keller

**Affiliations:** 1 Department of Ecology and Evolution, University of Lausanne1015, Lausanne, Switzerland; 2 Institute of Zoology, Zoological Society of LondonRegent's Park, NW1 4RY, London, UK; 3 School of Life Sciences, University of SussexBN1 9QG, Brighton, UK; 4 Laboratory of Developmental Genetics and Imprinting, The Babraham InstituteBabraham, CB22 3AT, Cambridge, UK

**Keywords:** Genetic structure, *Polistes canadensis*, *Polistes dominulus*, population viscosity, sociality, tropical/temperate

## Abstract

Climate has long been suggested to affect population genetic structures of eusocial insect societies. For instance, Hamilton [*Journal of Theoretical Biology* **7** (1964) 17] discusses whether temperate and tropical eusocial insects may show differences in population-level genetic structure and viscosity, and how this might relate to differences in the degree of synchrony in their life cycles or modes of nest founding. Despite the importance of Hamilton's 1964 papers, this specific idea has not been tested in actual populations of wasps, probably due to the paucity of studies on tropical species. Here, we compare colony and population genetic structures in two species of primitively eusocial paper wasps with contrasting ecologies: the tropical species *Polistes canadensis* and the temperate species *P. dominulus*. Our results provide important clarifications of Hamilton's discussion. Specifically, we show that the genetic structures of the temperate and tropical species were very similar, indicating that seasonality does not greatly affect population viscosity or inbreeding. For both species, the high genetic differentiation between nests suggests strong selection at the nest level to live with relatives, whereas low population viscosity and low genetic differentiation between nest aggregations might reflect balancing selection to disperse, avoiding competition with relatives. Overall, our study suggests no prevalence of seasonal constraints of the life cycle in affecting the population genetic structure of eusocial paper wasps. These conclusions are likely to apply also to other primitively eusocial insects, such as halictine bees. They also highlight how selection for a kin structure that promotes altruism can override potential effects of ecology in eusocial insects.

## Introduction

The general theoretical framework for the evolution of eusociality is provided by the concept of inclusive fitness theory (Hamilton 1964a,b), which states that individuals can pass on their genes to the next generation not only through their own reproduction but also indirectly through reproduction by relatives. The key components expected to influence the evolution of reproductive altruism are the relatedness between interacting individuals, and the impact of ecology of this. Differences in relatedness may arise either from individuals being close relatives (e.g., siblings) or population structuring with individuals being genetically more similar to individuals in their deme than individuals in other demes. Colony and population-level genetic structure may also be influenced by ecology. In his seminal paper, Hamilton discussed how the potential interactions of population structure, ecology (specifically, differences in seasonality of temperate and tropical climes), and life-history traits may influence the kin structure required for altruism. Yet, these factors remain little studied as comparative data on closely related species with contrasting ecologies (particularly tropical species) are limited.

In social insects, partitioning of genetic variance is generally organized at the level of the colony. However, life-history traits also influence higher level structuring between colonies, from a local scale (e.g., sub-populations) to larger scale population-level structuring. For example, in many species, there is limited dispersal by one of the sexes, which leads to isolation by distance with greater genetic similarity among individuals in colonies close together than in colonies further apart. In ants, where most studies of genetic structure in social insects have been performed, there appears to be a strong association between certain life-history traits, such as the number of breeders per colony or mode of dispersal, and the degree of genetic differentiation among colonies (Pamilo and Rosengren 1984; Ross and Keller 1995; Seppä and Pamilo 1995; Ross et al. 1997). In species with a single reproductive queen per colony (monogyny), young queens typically depart on a mating flight and initiate a new colony on their own after mating. By contrast, in species containing several queens per colony (polygyny), the young queens frequently return to an established colony after mating (Hölldobler and Wilson 1990; Keller 1991). In these species, new colonies are frequently initiated by budding, a process whereby queens leave their parental nest with workers to initiate a new colony nearby (short-ranged dispersal). Several studies have revealed that limited dispersal of queens in polygynous species is frequently associated with significant population viscosity (Pamilo and Rosengren 1984; Seppä and Pamilo 1995; Chapuisat and Keller 1999; Giraud et al. 2000; Liautard and Keller 2001; Fournier et al. 2002; Rüppell et al. 2003; Zhu et al. 2003; Zinck et al. 2007; Seppä et al. 2009; Rees et al. 2010). By contrast, the long-range dispersal of females in monogynous species usually leads to no significant population viscosity (Chapuisat et al. 1997).

In contrast to ants, we know little about how different modes of dispersal and colony founding influence population structure in eusocial wasps. However, the potential impact of ecology/climate may be greater in wasps, because they build short-lived annual colonies rather than long-lived perennial ones. Among the eusocial *Polistinae* wasps, modes of dispersal and colony founding may influence the breeding system (Pamilo et al. 1997). Independent-founding species (e.g., *Polistes*) are mostly monogynous (Reeve et al. 1991), whereas swarm-founding species, which form new nests by colony fission (e.g., *Polybia*), are usually highly polygynous (Jeanne et al. 1991; Pamilo et al. 1997). However, dispersal and modes of colony founding might also be associated with ecology, specifically climate (Reeve et al. 1991; Ross et al. 1991). Hamilton discussed how the interaction of climate (seasonality), life-history traits, and population structure might affect the conditions under which altruism can evolve in *Polistes* wasps, where temperate species tend to initiate new colonies without workers, whereas tropical species are more likely to initiate colonies “by swarms” (Hamilton 1964b; West-Eberhard 1969).

Here, we provide the first attempt to address this specific discussion by Hamilton. We aimed to compare at a microscale the population genetic structure of *Polistes dominulus* and *P. canadensis*. These two species of primitively eusocial wasps share many social traits in having a single egg-laying queen and high within colony relatedness, but they differ in their modes of colony founding and colony synchrony. *P. dominulus* is native to Europe, Asia, and North Africa (Judd and Carpenter 1996), where its colony cycle is constrained by the seasonality characterizing a temperate climate. Newly, singly mated gynes hibernate in communal shelters of sometimes hundreds of individuals to overcome the harsh winter conditions (Reeve et al. 1991; Dapporto et al. 2004). In the spring, the overwintered gynes then disperse and associate with a small number of other females to found new colonies (West-Eberhard 1969; Dapporto et al. 2004). It has been suggested that *P. dominulus* reproductives tend to be philopatric (Starks 2003; Dapporto et al. 2004), and indeed Hamilton suggested that there would be little effect of diapause on the relatedness of temperate co-foundresses, because they are often observed returning to their natal colony site before co-founding. However, recent genetic studies have revealed considerable variation in colony kin structure among *P. dominulus* co-foundings, with associations containing both related and unrelated females (Queller et al. 2000; Zanette and Field 2008; Leadbeater et al. 2010). These individuals sometimes lay eggs (Leadbeater et al. 2011), which should influence the degree of within-nest relatedness, as documented in several ant species with perennial colonies (Chapuisat et al. 1997; Liautard and Keller 2001; Zinck et al. 2007).

In contrast, nests founded by the tropical species *P. canadensis* in Panamá generally comprise large associations of females (Pickering 1980), and nest founding takes place throughout the year without a seasonally enforced diapause (Giray et al. 2005). This lack of seasonality may induce asynchronous male production, which may lead to some inbreeding because virgin females may be forced to mate with related males from the colony if colonies are relatively isolated, or if no other males are available from neighboring nests. Colony-level relatedness appears to be similarly high as *P. dominulus* (Sumner et al. 2007), and observations suggest that this probably stems from groups of sisters initiating new colonies close to the parental nests, not dissimilar to nest founding by budding in ants (West-Eberhard 1969; Pickering 1980). These life-history traits may lead to some population viscosity and genetic structuring at a relatively small scale. However, in contrast to temperate *Polistes*, there is currently very little genetic data on colony and population structure in tropical *Polistes* (Sumner et al. 2007). Thus, it is not yet clear to what extent (if any) these differences in ecology and life-history traits influence population structure and hence the conditions for altruism.

To test the hypothesis that differences in the mode of colony founding and colony synchrony between tropical and temperate colonies should affect the genetic structure of wasp populations, we therefore carried out a comprehensive analysis of genetic structure of both *P. canadensis* and *P. dominulus* populations.

## Methods

### Sampled populations

#### Polistes canadensis

**Figure 1:**
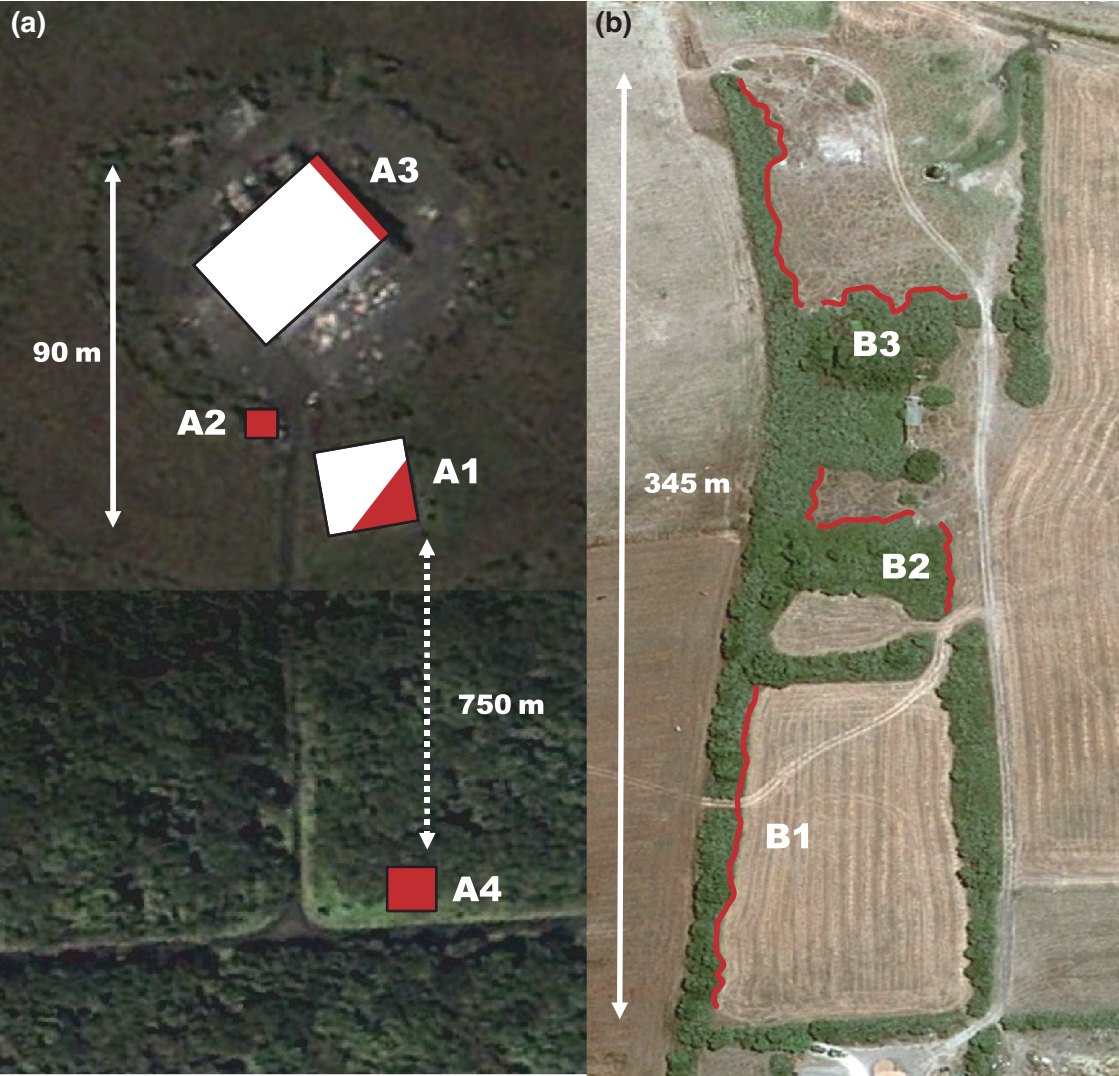
(a) Map of the sampling sites of *Polistes canadensis* located in Punta Galeta, Colón, Republic of Panamá (9°24′08.28″N, 79°52′19.41″O). Each rectangle or square corresponds to an aggregation. The areas shaded in red represent the distribution of studied nests within the aggregations (A1, A2, A3, and A4). Between A1 and A4, the entire surface area is not shown (for distances see dotted arrow) (b) Map of the sampling sites of *P. dominulus* located around Conil de la Frontera, Cádiz Province, Spain (36°15′10.76″N, 6°03′56.48″O). The red «lines» represent the distribution of studied nests along the hedges of *Opuntia* cacti. Adjacent «lines» form each of the three aggregations (B1, B2, and B3) (images from Google Earth).

In July 2009, twenty-six mature post-emergence (after the emergence of the first batch of workers) colonies were collected from a large natural population on abandoned buildings of over 200 nests located in Punta Galeta, Colón, Republic of Panamá (9°24′08.28″N, 79°52′19.41″O, under ANAM permit #SE/A-33-99, Fig. 1a). The population was subdivided into four aggregations (A1, A2, A3, and A4) in a series of abandoned buildings, each composed of 6–10 nests. Within aggregations, nests were separated by 40.1–681.4 cm (mean ± SD, 204.2 ± 176.3 cm). Aggregations were separated from each other by approximately 25 m (A1–A2) to 830 m (A3–A4). All females were individually marked and monitored in order to determine the queen's identity and exclude the possibility of queen turnover. All collected individuals were stored in 95% ethanol for later DNA analyses.

#### Polistes dominulus

Twenty-six randomly selected nests were sampled from a large population of over 200 nests in March 2008 around Conil de la Frontera, Cádiz Province, Spain (36°15′10.76″N, 6°03′56.48″O, Fig. 1b). All colonies were on hedges of *Opuntia* cacti. The population was subdivided into three aggregations (B1, B2, and B3), in a series of cacti banks, each containing from seven to ten nests. The distance between nests within aggregations ranged from 43.0 to 1047.9 cm (mean ± SD, 323.1 ± 247.7 cm). The mean distance between aggregations (measured from the center point of each aggregation) varied from 80 to 170 m. All females were individually marked and monitored in order to determine the queen's identity and exclude the possibility of queen turnover. All brood and adults were stored in 95% ethanol.

### Molecular Methods

#### Development of markers for *Polistes canadensis*

Previous genetic analyses on *P. canadensis* were performed using non-specific markers developed from various related species of *Polistes* (*P. annularis, P. bellicosus*, Strassmann et al. 1997; see Sumner et al. 2007). To increase the accuracy of our relatedness and genetic differentiation estimates, we developed species-specific microsatellites markers for *P. canadensis*. Seven polymorphic microsatellite loci were isolated and used in this study (Table 1). The first steps of microsatellite primers development, from DNA extraction to sequencing, were conducted by Ecogenics GmbH (Zurich, Switzerland) based on specimens collected in 2008 in the area of the Panamá City, Republic of Panamá (8°54′17.42″N, 79°34′35.41″O)(under ANAM permit #SE/A-53-08). DNA extractions were performed using one leg per individuals from each of 30 different nests. An enriched library was developed from size selected genomic DNA ligated into SNX forward/SNX reverse-linker (Hamilton et al. 1999) and enriched by magnetic bead selection with biotin-labeled (CT)_13_, (GT)_13_, (AAC)_10,_ and (AAG)_10_ oligonucleotide repeats (Gautschi et al. 2000a,b). Of 528 recombinant colonies screened, 367 gave a positive signal after hybridization. Plasmids from 36 positive clones were sequenced. Primers were designed from 22 inserts using the PRIMER SELECT module in the DNAStar computer program from Lasergene (DNASTAR Inc., Madison, WI), optimized and tested for polymorphism. From the initial 22 designed primers, we then selected seven highly polymorphic loci (number of alleles > 5) that yield clear amplification products (Table 1).

**Table 1:** Characterization of seven polymorphic loci in *Polistes canadensis*, including locus names and Genbank Accession no., repeat motifs (core repeats), size of cloned alleles, optimal annealing temperatures (Ta), optimal numbers of cycles and primer sequences

Locus	Genbank acc. no	Core repeats	Size(bp)	Na	T°a	Cycle	H_e_/H_o_	Primer seq. (5′-3′)	N	Used
Pcan01	JQ773392	(TTC)_9_	153	7	55	35	0.81/0.91	F: TCTTCTGAGCGTGTAAGTATCGTC R: CCAATTAATAGCCATAATTCAAAATG	129	YES
Pcan05	JQ773388	(CT)_11_	194	8	57	25	0.63/0.54	F: GATGGCTCGGTTCTTCTCT R: CAGGGAACTTTCGGTGTAA	129	YES
Pcan09	JQ773391	(GAA)_8_	128	5	57	30	0.62/0.59	F: CAGAAGAAGGGGGAGGTGACGAA R: CTGCGGATGAAGAGAAACGATGTG	129	YES
Pcan15	JQ773397	(GAA)_9_	281	9	55	30	0.79/0.80	F: TGTGTAGGAGAAAGAGGTATTG R: TATATTTTCCAAGTGATTTGTTG	129	YES
Pcan16	JQ773398	(GA)_48_	155	17	60	30	0.84/0.79	F: ATCAAGAGTTAGTAAAAGGGATAC R: GCACAGAATTGCAACTAAAC	129	YES
Pcan23	JQ773404	(AG)_22_	160	11	55	35	0.81/0.78	F: CACTTTGTAGGCTGGACGAT R: CGGAAGTGTAATAAACGAAATG	129	YES
Pcan24	JQ773405	(GT)_10_	194	7	57	30	0.80/0.81	F: TCTGTCCGATCTTCTGAACC R: AAGCGACCTGACATTGAATC	129	YES
Pcan03	JQ773386	(TTG)_2_ GTG (TTG)_7_	238	1	–	–	–	F: GAGGTTGCCGGACTGTGTTTTC R: TACATTCAATGGCAGAACGGAGTC	10	NO
Pcan04	JQ773387	(AAC)_15_	233	0	–	–	–	F: GAGGAGGAGGTGGAGGAAGTGGT R: CTGCTGCTGCTGTTGGTGATGA	10	NO
Pcan06	JQ773389	(GA)_30_	213	0	–	–	–	F: GGAAAAGGAAGGATGCATGTATGC R: CGACAGTTTTTCGCGGATCTTC	10	NO
Pcan08	JQ773390	(AAC)_3_ AAA (AAC)_7_	202	2	–	–	–	F: AGTGCCATATCTCAATTCGTCGTC R: GCAGCCGAACAACAAACAAGATAT	10	NO
Pcan11	JQ773393	(AAC)_2_ ACC (AAC)_2_ ATC (AAC)_6_	190	0	–	–	–	F: CATCAACAGCAGCAACAGCATCAC R: CAGCCGTGGTGGCGAGATTAC	10	NO
Pcan12	JQ773394	(CTT)_4_ CTA (CTT)_2_ CCA (CTT)_11_	130	1	–	–	–	F: TGACTGACTGCTTCCATCTCTT R: AGTGGCTCCCGAGGTAAAG	10	NO
Pcan13	JQ773395	(AAG)_2_ GAAA (AAG)_3_… (AAG)_5_	119	1	–	–	–	F: GAAAGGGCGAAACGACGTGAA R: AAAGTTCGTTTGCTTGTTCGTTCC	10	NO
Pcan14	JQ773396	(TC)_10_	258	2	–	–	–	F: TTCAAATGAAAAGAAATCAAGAAA R: GTAAATGAGAATAAGTGGCTGGAC	10	NO
Pcan17	JQ773399	(CT)_26_	165	0	–	–	–	F: CTCACATTCGGTTATCAGAAAT R: TTCGATTCGTTGAGAAGATG	10	NO
Pcan18	JQ773400	(AG)_16_ GG (AG)_18_	140	0	–	–	–	F: GAGAAAGACGGAAAACGTTTGAAG R: ATGAAGATGCACAGTGGATTTCTC	10	NO
Pcan19	JQ773401	(GT)_12_	266	0	–	–	–	F: CGCAGCGTGTTGAATGAATA R: ATGGGGATACAAAAGGAAACTAAG	10	NO
Pcan21	JQ773402	(GA)_15_ GG (GA)_5_	127	0	–	–	–	F: AGTGGTAGTAGGCAGGGAAAGAGG R: CGTCGCTCGTGCAACCTATTA	10	NO
Pcan22	JQ773403	(CA)_16_	233	3	–	–	–	F: TGCATGGACCAACGCTTATCTT R: TTGGGGTGAGGACGAAACATTA	10	NO
Pcan25	JQ773406	(TC)_14_	106	0	–	–	–	F: TGAATTTACGCACTGACACATC R: TCATAAGCAAAAGGACACAGACTA	10	NO
Pcan26	JQ773407	(TC)_19_	160	0	–	–	–	F: GGTCGTGCCAGGAAGAGAAG R: CAGCCGTTGTGGAAGAGGTC	10	NO

The number of alleles (Na) and observed and expected heterozygosities (He/Ho) are reported for a pooled sample of 129 individuals. The last column indicates whether the primer was used for the present genetic analysis. When Na = 0, no clear amplification was obtained after several runs.

### Genotyping methods

#### Polistes canadensis

As high levels of nests drifting by workers has been documented in the species (Sumner et al. 2007), some workers may not be individuals originating from the nest, but simply visitors from other colonies. This would potentially reduce colony-level relatedness, if we inadvertently sampled drifters from other colonies. Therefore, our relatedness estimates were based on the genotypes of late stage larvae and pupae. DNA was extracted from five pupae or large larvae (only females) from each of the 26 nests. Extraction and purification were carried out with BioSprint96 (Qiagen, Hombrechtikon, Switzerland) using the “Animal Tissue DNA Purification” kit and DNA analyzed at the seven polymorphic loci (Table 1). In total, 129 individuals were genotyped. PCR (polymerase chain reaction) amplifications were performed separately for each locus in 20 μL final volume containing 2 μL of DNA extract, 2 μL of (Qiagen) PCR buffer ×10, 2 μL of (Qiagen) Q-solution, 0.24 μL of dNTPs (2 mM), 11.56 μL of milli-Q water, 0.2 μL of *Taq* polymerase (Qiagen), and 1 μL of each *forward* (fluorescently labeled) and *reverse* primers (each 10 μM). Amplifications were conducted in a GeneAmp PCR System 9700 (Applied Biosystems, Foster City, California). The PCR mix was denatured at 95°C for 5 min and cycled 25–35 times, depending on the locus, at 95°C for 30 sec, at primers annealing temperature (55–60°C) for 30 sec and elongated at 72°C for 30 sec. A final elongation step at 72°C for 10 min followed to complete extension of PCR fragments. Labeled PCR products were analyzed on an ABI PRISM 3100 Genetic Analyser (Applied Biosystems) and allele sizes estimated using GENEMAPPER software (Applied Biosystems). Individuals that showed heterozygous alleles for at least one locus were considered as females; individuals that did not fit this criterion were considered as males and excluded from the analysis.

#### Polistes dominulus

DNA was extracted from five pupae or large larvae (only females) from each of the 26 nests. We used a selection of the primer sets previously isolated from *P. dominulus* (Pdom1jc, Pdom2jc, Pdom7, Pdom20, Pdom25jc, Pdom122jc, Pdom127b, Pdom140) (Henshaw 2000; Leadbeater et al. 2010) and *P. bellicosus* (Pbe128TAG) (Strassmann et al. 1997). In total, 129 individuals were genotyped. Multiplex PCR were carried out on a Peltier Thermal Cycler. Amplifications in a single multiplex mix were performed in 4 μL, containing approximately 80 ng of template DNA, 0.75 μM of the four primer pairs (Pdom1jc, Pdom2jc, Pdom20, Pbe128TAG), 0.375 μM of the remaining five primer pairs (Pdom7, Pdom25jc, Pdom122jc, Pdom127b, Pdom140), and 2 μL of PEQlab hot start mix Y (details in Leadbeater et al. 2010). A droplet of mineral oil was added to prevent evaporation. Multiplex mix was denatured at 95°C for 15 min and cycled 35 times at 94°C for 30 sec, 57°C (annealing temperature) for 90 sec, and 72°C for 60 sec. Final extension was performed at 60°C for 30 min. PCR products were separated by size using a 48-well capillary ABI 3730 sequencer (Applied Biosystems) and visualized using GENEMAPPER software (Applied Biosystems). For further genotyping information, see Leadbeater et al. 2010.

### Genetic analyses

#### Population genetic structure

Expected and observed heterozygosities were estimated using the program GDA 1.0 (Genetic Data Analysis, Lewis and Zaykin 2001). We tested linkage disequilibrium between pairs of loci and deviations from Hardy–Weinberg equilibrium randomizing 10000 times alleles among individuals within nests using the software GENEPOP 4.0.10 (Raymond and Rousset 1995). To account for the non-independence of nestmate genotypes, a resampling procedure providing unbiased estimates was performed for calculations of deviations from Hardy–Weinberg equilibrium and linkage disequilibrium. An R script was written to randomly select a single individual's multi-locus genotype from each nest and create 1000 distributions of independent genotypes. Iterations for each test, using the resampled genotype distributions, were performed using the “batch mode” option, available in the command line version of the GENEPOP software.

Genetic differentiation was quantitated for the different levels of biological organization (individual, nest, aggregation) by conducting a three-level hierarchical F-analysis. We measured Wright's hierarchical F-statistics, using Weir and Cockerham's (1984) method implemented in GDA 1.0. Five thousands bootstrap procedures were performed to give 95% confidence intervals. We also used ARLEQUIN (Excoffier et al. 2005) to provide significance for the F-statistics using the non-parametric permutation procedures implemented in the software. In the analyses, nests (_NEST_) and aggregations (_AGG_) were considered as two different levels of sub-populations and all sampled individuals as total populations (_TOTAL_). We estimated F-statistics at different levels: F_NEST-AGG_ (estimates of genetic differentiation between nests within aggregations)_,_ F_AGG-TOTAL_ (estimates of genetic differentiation between aggregations)_,_ F_IND-TOTAL_ (inbreeding coefficient of individuals relative to the total population).

#### Isolation by distance

Genetic differentiation (F_S_ = F_NEST-TOTAL_) between all pairs of nests was estimated using the software FSTAT 2.9.4 (Goudet 1995). To investigate patterns of isolation by distance, we plotted the transformed genetic distance formula F_ST_/(1 − F_ST_) against the natural logarithm of geographic distances, as proposed by Rousset (1997). Significance of the correlation between genetic and geographic distances was assessed with a Mantel test, implemented in FSTAT 2.9.4 (10000 permutations). We also examined isolation by distance within each aggregation using all nests in the aggregations as the total population for the calculations of pairwise F_ST_.

#### Colony genetic structure

Genetic relatedness (*r*) was also calculated within nests and within aggregations for the two populations of *Polistes* species using Queller and Goodnight's (1989), which is based on Grafen's (1985) relatedness coefficient. Calculations were performed using the program RELATEDNESS 5.0.8 by weighting nest equally. Standard errors were estimated by jackknifing over loci.

## Results

The number of alleles per locus in *P. canadensis* ranged from 5 (Pcan09) to 17 alleles (Pcan16) with a mean of 8.7 alleles (Table 1). The expected heterozygosities ranged from 0.63 to 0.84 (H_e_, all loci: 0.76), whereas the observed heterozygosities ranged from 0.54 to 0.91 (H_o_, all loci: 0.75). In *P. dominulus*, the number of alleles ranged between 5 (25jc) and 36 (Pdom122jc) with a mean of 13.8 alleles. The observed heterozygosities ranged from 0.53 to 0.99 (H_o_, all loci: 0.77) and the expected heterozygosities from 0.63 to 0.96 (H_e_, all loci: 0.79).

No significant departure from Hardy–Weinberg equilibrium was found in either *P. canadensis* or *P. dominulus* populations (all population, all loci: *P* > 0.05 for all 1000 resampled distributions). Additionally, for both species, we found no evidence of linkage disequilibrium between any pair of loci within populations using Fisher's exact test in GENEPOP. In *P. canadensis*, linkage disequilibrium was detected across four pairs of loci (over 21) in only 6% of the 1000 resampled dataset (max: 3.2% between Pcan16 and Pcan24). In *P. dominulus*, eight pairs of loci (over 36) showed significant linkage disequilibrium in only 8.4% of the 1000 resampled dataset (max: 4% between Pdom7 and Pdom140). All loci were then considered behaving as neutral markers and were kept for the analyses of genetic structure.

### Population genetic structure

The hierarchical analysis of population structure revealed high genetic differentiation between nests within *P. canadensis* aggregations (F_NEST-AGG_ = 0.359, *P* < 0.0001; L-95% CI: 0.326; U-95% CI: 0.386). A weaker but significant genetic differentiation was also found between aggregations (F_AGG-TOTAL_ = 0.023, *P* = 0.016, L-95% CI: 0.008; U-95% CI: 0.037). There was no evidence of inbreeding at the population level (F_IND-TOTAL_ = 0.032, *P* = 0.15; L-95% CI: -0.024; U-95% CI: 0.087), suggesting that mating occurs randomly (see Table 2).

**Table 2:** Estimates of inbreeding, genetic differentiation with confidence intervals in *Polistes canadensis* and *P. dominulus*, and *t*-test significance between species

	F_IND-TOTAL_	F_NEST-AGG_	F_AGG-TOTAL_
*P. canadensis*			
All Loci	0.032 ns	0.359***	0.023*
95% CI L:	−0.024	0.326	0.008
U:	0.087	0.386	0.037
*P. dominulus*			
All Loci	0.043**	0.332***	0.005 ns
95% CI L:	−0.006 0.093	0.310 0.353	−0.005 0.013
U:	0.093	0.353	0.013

Asterisks represent significance of randomization tests performed in Arlequin.

*** *P* < 0.001.

** *P* < 0.01.

* *P* < 0.05, ns = non-significant.

In *P. dominulus*, the hierarchical analysis of population structure indicated a strong genetic differentiation between nests within aggregations (F_NEST-AGG_ = 0.332, *P* < 0.0001; L-95% CI: 0.310; U-95% CI: 0.353), but no significant genetic differentiation between aggregations (F_AGG-TOTAL_ = 0.005, *P* = 0.22; L-95% CI: −0.005; U-95% CI: 0.013). The value F_IND-TOTAL_ was low, but significantly greater from zero (0.043, *P* = 0.008; L-95% CI: −0.006; U-95% CI: 0.093), indicating a low level of inbreeding (see Table 2).

Pairwise comparisons between nests showed no significant isolation by distance in *P. canadensis* when nests of the four aggregations were considered simultaneously (Mantel tests, *r* = 0.157; *P* = 0.11; 10000 permutations). There was, however, a significant isolation by distance in one of the four aggregations (aggregation A3, *r* = 0.895; *P* = 0.027). In the three other aggregations, there was also a slight positive correlation, but it was not significant (A1 *r* = 0.011, *P* = 0.94; A2, *r* = 0.053, *P* = 0.77; A4, *r* = 0.272, *P* = 0.45). Interestingly, aggregation A3 differed from the three others in that nests were located in a relatively open space, whereas nests were clustered inside buildings in the three other aggregations.

In the Spanish population of *P. dominulus*, there was a low but significant isolation by distance when considering all nests of the three aggregations (*r* = 0.131; *P* = 0.024). An analysis of each aggregation separately revealed significant isolation by distance in B2 *r* = 0.330, *P* = 0.014, and B3 (*r* = 0.332, *P* = 0.027), but not in B1 (*r* = 0.009; *P* = 0.97). Although we found only significant isolation by distance in *P. dominulus*, estimations of isolation by distance at both the population level and for each aggregation were relatively low and did not seem to fundamentally differ between the two species.

The mean within-nest relatedness was high (and not significantly different) for both species (*P. canadensis*: *r* = 0.69 ± 0.02; *P. dominulus*: *r* = 0.64 ± 0.01, two-tailed *t*-test: *P* > 0.05) and close to the theoretical relatedness values between haplodiploid full-sisters (*r* = 0.75). Genetic relatedness at the aggregation level were significantly greater than zero in both species, and higher in *P. canadensis* (*r* = 0.12 ± 0.01) than *P. dominulus* (*r* = 0.07 ± 0.01, two-tailed t-test: *P* = 0.006). This suggests a higher degree of population structuring in *P. canadensis* relative to *P. dominulus*, which parallels the conclusion from our complementary F-statistics analyses.

## Discussion

The result of our genetic study provides no support for Hamilton's statement that the genetic structuring of populations may differ between tropical and temperate areas, at least for the studied species. Overall, there were only small differences in the population genetic structure of *P. canadensis* and *P. dominulus*.

At the colony level, there was a high relatedness among female offspring in both species (*P. canadensis*: *r* = 0.69 ± 0.02; *P. dominulus*: *r* = 0.64 ± 0.01), suggesting that most colonies are headed by one singly mated queen. These results are consistent with previous studies performed on *P. canadensis* and *P. dominulus*, which showed, through observations or genetic analyses, an almost exclusive monopoly of the reproduction by the queen (*P. canadensis* (West-Eberhard 1969; S.S unpublished data) and *P. dominulus* (Zacchi 1998; Queller et al. 2000; Zanette and Field 2008)). Moreover, contrary to Hamilton's predictions, our colony genetic structure analysis revealed no pronounced level of inbreeding in the tropical species *P. canadensis*. Indeed, we found some evidence of the opposite, with a low level of inbreeding in the temperate species *P. dominulus*.

Our study also revealed only limited population structuring at levels higher than the nest in both species. In *P. canadensis*, the level of genetic differentiation between aggregations was very small (F_AGG-TOTAL_ = 0.023), whereas in *P. dominulus*, no significant differentiation was detected. Consistent with these findings, there was also a very limited isolation by distance in both species.

In *P. canadensis*, we found no isolation by distance at the level of the population, or in three of the four aggregations studied. Interestingly, however, there was a significant structuring within one aggregation (A3: *r* = 0.895). In contrast to the three other associations, which were located (at least partially) within buildings, the aggregation A3 was in a more open area (overhangs of building). This suggests that topography of the nesting sites may influence the distribution of nests and the dispersal behavior of individuals. It would be of interest to compare populations of *P. canadensis* within buildings and populations in natural habitat (e.g., trees, open areas) to investigate whether patterns of dispersal are generally influenced by differences in topography.

These results of population structure found in *P. canadensis* are particularly interesting with regard to a recent study, which revealed high levels of nest drifting between closely located nests in a Panamanian population of *P. canadensis* (Sumner et al. 2007). This study suggested that visiting individuals may gain indirect fitness benefits by helping raise the brood in closely located nests if nests are genetically similar (viscous population). This hypothesis seems unlikely in light of the finding of low population structuring and absence of isolation by distance in that species. Although no isolation by distance was detected in the *P. canadensis* population, nests within aggregations seem on average related (*r* = 0.12 ± 0.01), which suggest that, even in non-viscous populations, drifters may still be able to benefit from indirect fitness by delivering help in neighboring nests. However, it is also possible that isolation by distance was present in past populations of *P. canadensis* and helped promoting the evolution of nest drifting, but disappeared over time due to the availability of human structures, which have altered their dynamics of dispersal.

Intriguingly, similar levels of indirect fitness could theoretically be achieved by nest drifting in *P. dominulus*, where there is a similar level of population structuring to *P. canadensis*. Nest drifting is difficult to detect using traditional manual censusing methods, as is currently practiced on *P. dominulus*. It is possible that finer level monitoring (e.g., using radio-frequency identification tags) would in fact detect nest drifting by workers for indirect fitness benefits in *P. dominulus*, if this behavior is a general phenomenon.

Unlike *P. canadensis*, there was a slight isolation by distance at the population level (*r* = 0.13) as well as population viscosity in two of the three aggregations in *P. dominulus*. This suggests limited dispersal in these species, with a significant fraction of females initiating new nests in the vicinity of their parental nest.

These results on colony and population genetic structure found in *P. canadensis* and *P. dominulus* were determined from data collected from small-scale populations (<1 km between aggregations). Significant micro-structuring has been reported in *P. exclamans*, another species of primitively eusocial wasps (Davis et al. 1990). In this temperate species, high levels of genetic structure were detected. By contrast, there was no evidence of genetic structuring at a micro-geographic scale in *P. bellicosus, P. carolinus, and P. metricus*, which are sympatric to *P. exclamans* (Davis et al. 1990).

The use of a small scale in our study was primarily due to the limited number of wasp aggregations for both *P. canadensis* and *P. dominulus*. Additional studies on populations at larger scale as well as in other tropical and temperate species are necessary to unambiguously reject the hypothesis of a difference in population structure between temperate and tropical species. Further investigations on population structure in social insects from different climates may also benefit by examining species living in both tropical and temperate areas. *P. dominulus* may be a suitable model for such comparison as its distribution is widespread across the world. Comparing genetic structure of subtropical populations (with no clear seasonality), into which the species range appears to be expanding naturally (Cervo et al. 2000), to native European populations may prove valuable for investigating the potential behavioral and genetic differences. However, one should be cautious because *P. dominulus* has been largely introduced in several countries, such as the US, Australia, and Chile (Judd and Carpenter 1996). Invasive populations may not reflect the same natural sets of genetic and behavioral features found in native populations, especially because of potential genetic bottlenecks, induced by independent introductions, which may lower the frequency distribution of alleles in the population (Luikart et al. 1998) and induce changes in the population genetic composition. So far, most genetic studies of populations of *P. dominulus* introduced in the US suggest no severe bottleneck with populations having high genetic variability (Johnson and Starks 2004; Liebert et al. 2006). However, other populations in the US have already showed behavioral modifications to nest founding (Liebert et al. 2006) as well as the presence of diploid males and triploid females (Liebert et al. 2005).

Overall, this study found genetic structure between temperate and tropical species of paper wasps to be far more similar than expected, considering the contrasts in life cycles and nest-founding behavior of *P. canadensis* and *P. dominulus*. Strong selection to nest with relatives was revealed by high structuring at the nest level. However, we found low population viscosity and low differentiation between aggregations in both species. This might reflect the effects of balancing selection in promoting emigration outside the natal aggregations and hence avoiding competition between close relatives (Hamilton and May 1977; Taylor 1988, 1992). Such small differences in population structure between the two species with contrasting ecology indicates that differences in climate with seasonal constraint on the life cycle may not to be such a fundamental factor affecting the genetic structure of populations. Thus, we provide some resolution to the discussions raised by Hamilton on the potential connections between modes of nest founding, seasonality, and population structure in his seminal paper on inclusive fitness theory (Hamilton 1964b). Future studies and comparisons with more tropical species are required to further confirm these insights, and to gain a better understanding of the impacts of contrasting life cycles and seasonality on the population genetic structure of insect societies.

## Data Accessibility

Microsatellite sequences: Genbank accession numbers JQ773386-JQ773407 (see Table 1).
